# A dyadic survey study of partner engagement in and patient receipt of guideline-recommended colorectal cancer surveillance

**DOI:** 10.1186/s12885-022-10131-3

**Published:** 2022-10-13

**Authors:** Christine M. Veenstra, Katrina R. Ellis, Paul Abrahamse, Kevin C. Ward, Arden M. Morris, Sarah T. Hawley

**Affiliations:** 1 University of Michigan, 300 North Ingalls, NIB, Room 3A22, 48109 Ann Arbor, MI USA; 2 Emory University, Atlanta, GA USA; 3 Stanford University, Stanford, CA USA

**Keywords:** Colorectal cancer, Surveillance, Dyadic, Partner

## Abstract

**Background:**

We investigated whether partner (spouse or intimate partner) engagement in colorectal cancer (CRC) surveillance is associated with patient receipt of surveillance.

**Methods:**

From 2019 to 2020 we surveyed Stage III CRC survivors diagnosed 2014–2018 at an academic cancer center, a community oncology practice and the Georgia SEER registry, and their partners. Partner engagement was measured across 3 domains: Informed about; Involved in; and Aware of patient preferences around surveillance. We evaluated bivariate associations between domains of partner engagement and independent partner variables. Analysis of variance and multivariable logistic regression were used to compare domains of engagement with patient-reported receipt of surveillance.

**Results:**

501 patients responded (51% response rate); 428 had partners. 311 partners responded (73% response rate). Partners were engaged across all domains. Engagement varied by sociodemographics. Greater partner involvement was associated with decreased odds of receipt of composite surveillance (OR 0.67, 95% CI 0.48–0.93) and trended towards significance for decreased odds of receipt of endoscopy (OR 0.60, 95% CI 0.34–1.03) and CEA (OR 0.75, 95% CI 0.55–1.04). Greater partner awareness was associated with increased odds of patients’ receipt of endoscopy (OR 2.18, 95% CI 1.15–4.12) and trended towards significance for increased odds of receipt of composite surveillance (OR 1.30, 95% CI 0.91–2.04).

**Conclusion:**

Partners are engaged (informed, involved, and aware) in CRC surveillance. Future research to develop dyadic interventions that capitalize on the positive aspects of partner engagement may help partners effectively engage in surveillance to improve patient care.

**Supplementary Information:**

The online version contains supplementary material available at 10.1186/s12885-022-10131-3.

## Background

Over 40% of patients with Stage III colorectal cancer (CRC) will experience a cancer recurrence after completion of curative-intent treatment [1]. Limited recurrence, or metastasis to a single organ site, can be treated for a “second chance” at cure, with a cure rate as high as 50% [2, 3]. CRC surveillance, therefore, is critical to identify recurrence early, during the potentially curable period [4]. Guideline-concordant surveillance includes a combination of laboratory testing for carcinoembryonic antigen (CEA), cross-sectional imaging, and colonoscopy at regular intervals for 3–5 years [4–6]. While effective, this regimen may be burdensome for patients. Indeed, over half of the 1.2million survivors of CRC in the United States fail to receive guideline-concordant surveillance [7, 8] and consequently may miss an opportunity for early detection and cure of recurrent cancer.

This gap in care mandates inquiry into modifiable patient- and family-level factors that influence receipt of surveillance. Understanding and leveraging the influence of informal support systems, particularly the spouses or intimate partners of patients with CRC (i.e., partners), may provide an opportunity to improve meaningful clinical outcomes. Approximately 60% of patients with CRC are married or in an intimate partner relationship [9] and patients’ and partners’ views about cancer are often interdependent, as evidenced by mutual influence on attitudes, health behaviors, and health outcomes [10–12]. Previous work has shown that partners participate in and contribute to decision-making around patients’ options for cancer treatment [13, 14]. Similarly, in qualitative work we found that partners report engagement in patients’ CRC surveillance across multiple domains [15]. Yet, it is unknown whether engagement varies by partners’ characteristics, and whether the level of partners’ engagement is associated with patients’ receipt of guideline-recommended surveillance. Thus, using a unique dataset consisting of dyadic survey data from survivors of Stage III CRC and their partners, we sought to understand the degree to which partners engage in CRC surveillance, explore variations in engagement by partner characteristics, and investigate associations between patient receipt of guideline-concordant surveillance and partner engagement.

## Methods

### Study population

We identified patients aged 21–85 with surgically resected, pathologic Stage III colon or rectal cancer diagnosed 2014–2018. Patients were identified via the tumor registries of the University of Michigan (Ann Arbor, Michigan) and the Billings Clinic (Billings, Montana), and via archival registry data from the Georgia Surveillance, Epidemiology, and End Results (SEER) registry. Exclusion criteria included metastatic cancer (Stage IV) at diagnosis, identifiable cancer recurrence in the years between completion of curative-intent therapy and receipt of survey, and death prior to survey deployment. Partners (spouse, domestic partner, significant other) living in the same household as the patient—as identified by the patient—were also eligible.

### Data collection

Between April 2019 and February 2020 we used a modified multimodal Dillman approach [16] to invite patients and partners to participate as done in multiple prior studies by our team [13, 17–19]. Eligible patients were mailed a large envelope containing both a patient survey packet with a $10 cash gift and a separate partner survey packet for the patient to give to their partner. Patients were asked to first complete their survey packet, including a question on whether they have a partner. Those without a partner completed a shorter survey. Those with a partner were asked to complete the full patient survey and give the partner survey packet to their partner to complete. Patients and partners returned their completed surveys in separate envelopes. Upon receipt, partners were mailed a $10 cash gift. Completed surveys from patients and their corresponding partners were linked using unique identification numbers. We performed extensive data checks of completed surveys for logic, errors, and omissions, and contacted participants as needed to obtain missing information. The study protocol was approved by the Institutional Review Boards of the University of Michigan, the Billings Clinic, Emory University, and the State of Georgia Department of Public Health. All study methods were carried out in accordance with relevant guidelines and regulations.

### Measures

The questionnaire content for both patients and partners was developed from a conceptual framework of couples dealing with cancer developed by Northouse et al. [20], and was informed by research by our team and others on the role of family and friends in decision making [21–24]. We used standard techniques to assess content validity, including expert reviews and cognitive pretesting and pilot testing of measures in selected populations.

### Primary outcome

Patient-reported receipt of surveillance. To assess patient-reported receipt of guideline-concordant CRC surveillance, patients were asked (1) whether they received cross-sectional imaging in the form of computed tomography (CT) scan, magnetic resonance imaging (MRI), or positron emission tomography (PET) scan in the past 12 months; (2) whether they ever had sigmoidoscopy or colonoscopy after completing cancer treatment; and (3) whether they had a blood test for carcinoembryonic antigen (CEA) within the past 12 months (all yes/no). Receipt of each element of surveillance was assessed individually. Because optimal surveillance is a “package” of care in which all elements are necessary and should be received, a composite measure (patient received all three elements of surveillance, yes/no) was also created.

### Key covariates

Partner Engagement. The measures of partner engagement were previously developed by our team in the context of breast cancer care [13] and were based on existing measures [21, 22, 25–27]. To ensure these measures were relevant to CRC, we conducted qualitative work with a sample of survivors of CRC and their partners to revise the wording. We then pilot-tested the CRC-specific measures with that same sample and incorporated their feedback into the final version of the measures. We asked partners about 3 domains of engagement in surveillance: (1) Informed, characterized as partners’ perceptions of being informed about specific aspects of patients’ surveillance; (2) Involved, divided into two sub-domains—partners’ extent of involvement in patients’ surveillance, and partners’ satisfaction with that involvement; (3) Aware, characterized by partners’ perceptions of being aware of patients’ underlying values and preferences around surveillance.

We used factor analysis, Cronbach’s α, and item response theory to assess each engagement domain. Table 1 shows the specific items, response scales, and Cronbach’s α for each domain. We measured the domain Informed using 4 items. Responses were tabulated as a count of the number of items for which partners responded that they have received enough information and were scored from 0 to 4, with higher scores indicating being more informed. We measured the domain Involved by asking partners to report on two sub-domains. Extent of involvement was measured using 8 items. Responses to these items were averaged to create a composite score with higher scores indicating greater involvement. Satisfaction with involvement was measured using 2 items. After reversing the scoring for the item about wanting to participate more to align the directionality of responses, responses to these items were averaged to create a composite score with higher scores indicating greater satisfaction. The domain Aware was measured using a single item. Responses to this item were converted to a numeric score from 1 to 5, with higher scores indicating greater awareness.

**Table 1:** Domains of Partner-Reported Engagement in Colorectal Cancer (CRC) Surveillance

Domain	Definition	Items	Cronbach’s α
Informed	Perception of being informed about the risks and benefits of surveillance care	Thinking about your partner’s follow-up care for CRC, do you feel that you have received enough information about: • Risks/benefits of follow-up care in general • Risks/benefits of follow-up imaging • Risks/benefits of follow-up colonoscopy/sigmoidoscopy • Risks/benefits of follow-up CEA blood tests	0.82
Involved	Extent of involvement in surveillance	Thinking about your partner’s follow-up care for CRC, how often do you: • Help take your partner to follow-up appointments • Attend doctor appointments for follow-up • Take notes during doctor appointment • Help schedule follow-up appointments • Help keep track of follow-up appointments on a calendar • Remind your partner about follow-up appointments • Talk to your partner about follow-up care options • Share information with your partner from other sources about follow-up care	0.90
	Satisfaction with involvement in surveillance	Please tell us how you feel about the following statements (5-point Likert scale from “Not at all” to “Very much”) • I am satisfied with the amount of involvement I have in my partner’s follow-up care • I would like to participate more in my partner’s follow-up care	0.62
Aware	Awareness of patients’ preferences for surveillance	How aware are you about your partner’s **preferences** for follow-up care (5-point Likert scale from “Not at all” to “Very”)	N/A

Other covariates. Partners reported their age, gender, race and ethnicity, educational attainment (high school or less, some college, college graduate), and comorbid health conditions (0, ≥ 1). At the patient level, because of expected co-linearity between partner and patient sociodemographic factors, only patient-reported annual household income (<$40,000, $40,000-$89,999, ≥$90,000) and relevant patient clinical factors were included in these analyses. Patients reported their comorbid health conditions (0, ≥ 1), primary cancer site (colon, rectal), and cancer treatment, including receipt of chemotherapy (yes/no) and radiation therapy (yes/no), as done in our prior work [28].

### Missing Data

In both partner and patient surveys there were few missing values (< 3%) for all variables except income, for which 13% of patients did not respond or reported they did not know. Multiple imputation techniques were used to account for the missing annual household income data [29, 30].

### Statistical analyses

We first performed descriptive analyses of partner-reported perceptions of their engagement in CRC surveillance. Using analysis of variance tests, we evaluated bivariate associations between each domain of partner engagement (informed, involved, aware) and independent partner variables. We then used analysis of variance and multivariable logistic regression to investigate associations between the domains of partner engagement with patient-reported receipt of surveillance. To reduce potential bias due to nonresponse, weights were created with a logistic regression of partner nonresponse on demographic characteristics of the patients and were used in the multivariable analyses. All statistical tests were 2-sided; P values < 0.05 were considered significant. Analyses were conducted with SAS 9.4 (SAS, CARY, North Carolina).

## Results

### Study cohort

**Fig. 1:**
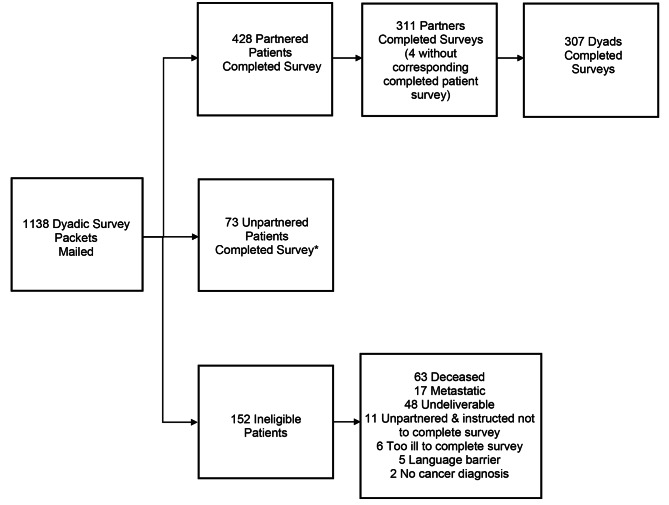
Flow of patients and partners into the study sample. *Patients recruited from the Georgia SEER registry were selected for survey mailing based on the indication that they were married/partnered as recorded in the registry data. Patients from the other study sites were mailed surveys without prior knowledge of their partnered status

Of 986 eligible patients, 501 returned surveys (51% patient response rate). Among those, 428 (85%) reported having a partner. Three hundred eleven partners returned surveys (73% partner response rate). Four partner surveys were returned without a corresponding completed patient survey; therefore, as shown in the study flow diagram (Fig. 1) paired surveys from 307 patient-partner dyads were included in these analyses. Patient response rates were significantly lower for non-White patients, male patients, and patients identified through the Georgia SEER site (all p < 0.05). Compared to patients with a partner, unpartnered patients were more likely to be female or Black (all p < 0.05).

### Patient and partner characteristics

88% of patients were > age 50, 64% were male, 85% were White, and 77% had at least some college education. 61% had a primary tumor in the colon, 95% received chemotherapy, and 34% received radiation. Most (62%) were diagnosed 3–4 years previously. Correspondingly, 84% of partners were > age 50, 63% were female, 86% were White, and 73% had at least some college education (Table 2).

**Table 2:** Characteristics of Patients (n = 307) and Partners (n = 307)

	Patients	Partners
**Characteristic**	**No. (%)**	**No. (%)**
Age, years		
≤50 51–64 ≥65	36 (11.8) 128 (41.8) 142 (46.4)	48 (16.2) 118 (39.9) 130 (43.9)
Gender		
Male Female	195 (63.7) 111 (36.3)	113 (37.4) 189 (62.6)
Race		
White Black Other	262 (85.3) 23 (7.5) 22 (7.2)	263 (85.7) 20 (6.5) 24 (7.8)
Education		
High school or less Some college College graduate	70 (22.8) 104 (33.9) 133 (43.3)	82 (27.0) 108 (35.5) 114 (37.5)
Annual Household Income		
<$40,000 $40,000-$89,999 ≥$90,000 Missing/Unknown	55 (17.9) 95 (30.9) 116 (37.8) 41 (13.4)	N/A
Comorbid conditions		
0 1 or more	87 (28.3) 220 (71.7)	78 (25.4) 229 (74.6)
Patients’ primary cancer		
Colon Rectum Both/unknown	187 (60.9) 40 (13.0) 80 (26.1)	N/A
Years since diagnosis		N/A
1–2 3–4 ≥5	78 (26.4) 182 (61.5) 36 (11.2)	
Patient receipt of chemotherapy		
Yes No	285 (94.7) 16 (5.3)	N/A
Patient receipt of radiation		N/A
Yes No	102 (34.0) 198 (66.0)	

### Patient-reported receipt of surveillance

Overall, 94% of patients reported receipt of cross-sectional imaging, 88% reported receipt of endoscopy, 55% reported receipt of CEA, and 42% reported combined receipt of all 3 elements of surveillance.

### Partner engagement

**Fig. 2:**
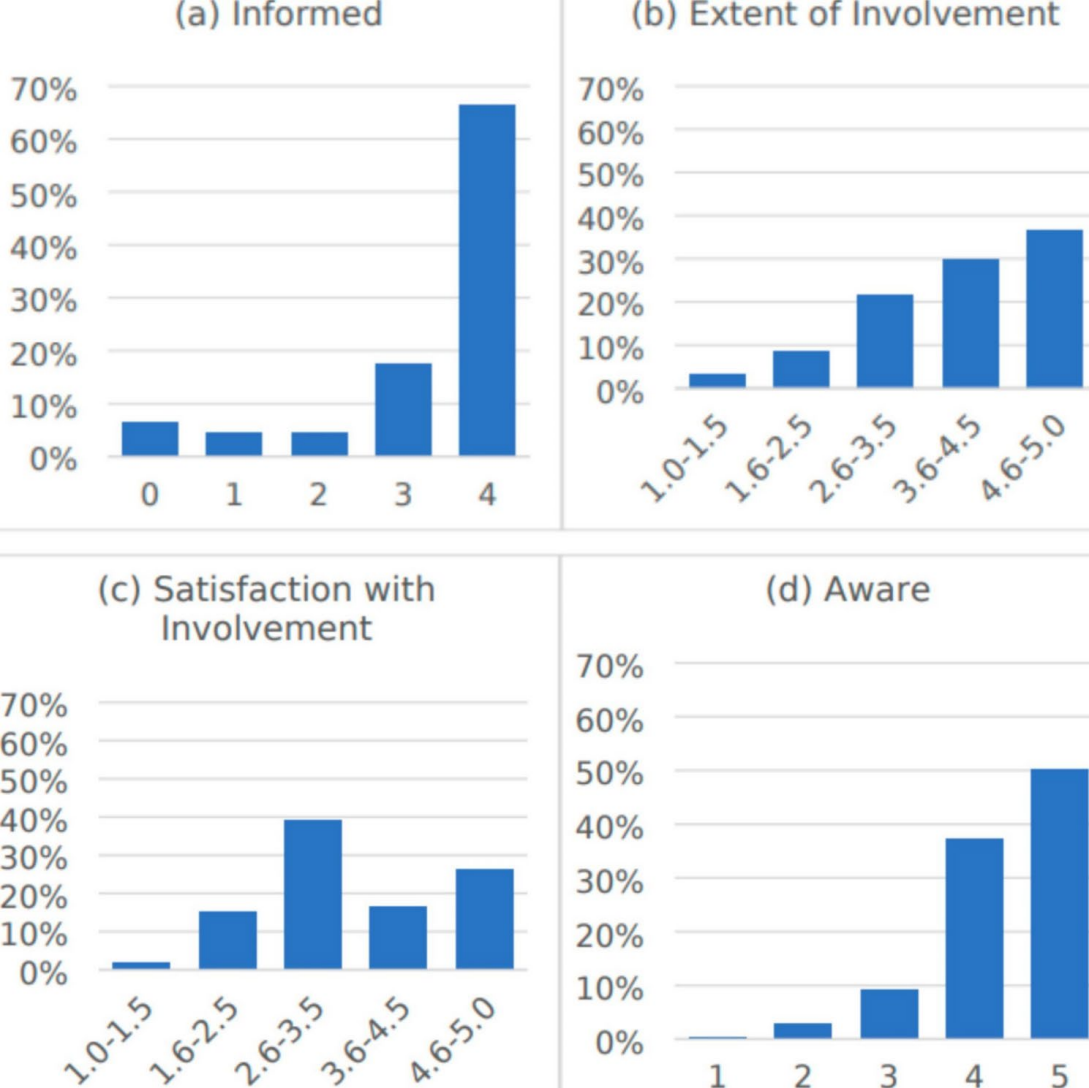
The distribution of scores for each domain of partner engagement is illustrated as follows: (**a**) Informed (range 0–4, mean score 3.33, standard deviation 1.18), (**b**) Extent of Involvement (range 1–5, mean score 3.58, standard deviation 1.03), (**c**) Satisfaction with Involvement (range 1–5, mean score 2.67, standard deviation 1.01), (**d**) Aware (range 1–5, mean score 4.34, standard deviation 0.79)

Informed. The mean score for informed was 3.33, with a range of 0–4 and standard deviation 1.18 (Fig. 2a). The proportion of partners who reported receiving enough information about each item are: surveillance in general (86%), imaging (86%), endoscopy (87%), CEA (74%) (Supplemental Fig. 1a). In bivariate analyses (Table 3), partners with a lower level of educational attainment were more likely to perceive being informed (p = 0.03).

**Table 3:** Bivariate Analyses of Domains of Partner-Reported Engagement

	Informed		Extent of Involvement		Satisfaction with Involvement		Aware	
**Partner Characteristic**	**Mean Score (Standard Deviation)**	**P**	**Mean Score (Standard Deviation)**	**P**	**Mean Score (Standard Deviation)**	**P**	**Mean Score (Standard Deviation)**	**P**
Age, years		0.06		0.80		0.02		0.21
≤50 51–64 ≥ 65	3 (1.46) 3.35 (1.1) 3.46 (1.08)		3.54 (1.05) 3.55 (1.04) 3.63 (1.04)		2.34 (1.09) 2.65 (1) 2.84 (0.98)		4.19 (0.82) 4.42 (0.8) 4.33 (0.77)	
Gender		0.72		< 0.01		0.15		< 0.01
Female Male	3.37 (1.13) 3.29 (1.23)		3.83 (0.99) 3.19 (1.01)		2.75 (1.04) 2.55 (0.97)		4.48 (0.68) 4.12 (0.91)	
Race		0.07		0.23		0.04		0.32
Black White Other	2.81 (1.6) 3.39 (1.11) 3.06 (1.48)		3.35 (1.38) 3.63 (1.02) 3.14 (0.87)		2.1 (1.12) 2.73 (1) 2.5 (1.08)		4.11 (1.08) 4.37 (0.76) 4.17 (0.92)	
Education		0.03		< 0.01		0.46		< 0.01
High school or less Some college College graduate	3.66 (0.76) 3.29 (1.18) 3.17 (1.33)		3.91 (0.95) 3.63 (1.01) 3.33 (1.07)		2.73 (0.96) 2.59 (1.03) 2.72 (1.05)		4.56 (0.66) 4.38 (0.78) 4.16 (0.85)	
Annual Household Income^a^		0.52		0.03		0.90		0.81
<$40,000 $40,000-$89,999 ≥$90,000	3.23 (1.31) 3.4 (1.11) 3.35 (1.14)		3.9 (1.02) 3.54 (1) 3.45 (1.06)		2.65 (1.08) 2.71 (0.96) 2.66 (1.04)		4.43 (0.8) 4.31 (0.79) 4.32 (0.8)	
Comorbid conditions 0	3.21 (1.22)	0.29	3.5 (1.1	0.52	2.77 (1.05)	0.15	4.36 (0.73)	0.90
1 or more	3.39 (1.14)		3.61 (1.02)		2.65 (1.01)		4.34 (0.82)	

^a^Multiple imputation was used to assign household income values for the 13% of respondents who did not report it

Involved. The mean score for extent of involvement was 3.58, with a range of 1–5 and standard deviation 3.33 (Fig. 2b). The items that partners most commonly reported doing very often were attending surveillance appointments (51%), taking patients to surveillance appointments (51%), and keeping track of surveillance appointments (47%). The items that partners most commonly reported never doing were taking notes during surveillance appointments (22%) and scheduling surveillance appointments (21%) (Supplemental Fig.1b). In bivariate analyses (Table 3), partners who were female (p < 0.01), had lower educational attainment (p < 0.01), and had annual household income <$40,000 (p = 0.03) more often reported a greater extent of involvement.

The mean score for satisfaction with involvement was 2.67, with a range of 1–5 and standard deviation 1.01 (Fig. 2c). 57% of partners reported being very satisfied with their involvement in surveillance, while 25% of partners desired more involvement in surveillance (Supplemental Fig.1c). In bivariate analyses (Table 3), partners who were younger (p = 0.02) and White (p = 0.04) were more likely to report greater satisfaction with involvement (p = 0.01).

Aware. The mean score for aware was 4.34, with a range of 1–5 and standard deviation 0.79 (Fig. 2d). 50% of partners reported being very aware of patients’ preferences for surveillance (Supplemental Fig.1d). In bivariate analyses (Table 3), female partners (p < 0.01) and partners with a lower level of educational attainment were more likely to report greater awareness of patients’ surveillance preferences (p < 0.01).

### Patient-reported receipt of surveillance and partner engagement

Informed. In bivariate analyses, partners’ perception of being informed about surveillance was significantly associated with an increased likelihood of patient receipt of surveillance endoscopy (p = 0.05). In multivariable analyses (Table 4), partners’ perception of being informed about surveillance was not significantly associated with patient receipt of any elements of surveillance.

**Table 4:** Multivariable Regression Models of Patient-Reported Receipt of Colorectal Cancer Surveillance

	Receipt of cross-sectional imaging	Receipt of endoscopy	Receipt of CEA	Receipt of all surveillance components
	**OR (95% CI)**	**OR (95% CI)**	**OR (95% CI)**	**OR (95% CI)**
**Partner Characteristics**				
Informed	0.96 (0.50–1.85)	1.12 (0.76–1.65)	1.10 (0.85–1.43)	0.95 (0.73–1.24)
Extent of involvement	1.23 (0.62–2.46)	0.60 (0.34–1.03)	0.75 (0.55–1.04)	0.67 (0.48–0.93)
Satisfaction with Involvement	0.92 (0.45–1.88)	1.07 (0.66–1.72)	1.17 (0.87–1.57)	1.28 (0.95–1.73)
Aware	1.46 (0.65–3.27)	2.18 (1.15–4.12)	0.83 (0.54–1.28)	1.30 (0.91–2.04)
Annual Household Income				
<$40,000	REF	REF	REF	REF
$40,000-$89,999	2.88 (0.62–13.30)	4.16 (1.19–14.59)	1.97 (0.92–4.21)	2.47 (1.07–5.70)
≥$90,000	1.47 (0.31–7.04)	1.36 (0.41–4.57)	2.08 (0.94–4.59)	2.03 (0.86–4.77)
**Patient Characteristics**				
Receipt of chemotherapy				
No	REF	REF	REF	REF
Yes	4.84 (0.74–31.66)	9.97 (1.78–55.70)	0.91 (0.23–3.64)	8.85 (0.98–80.08)

95% CI: 95% confidence interval. Models also adjusted for partner age, gender, race, education, comorbid conditions and patient comorbid conditions, site of primary cancer, years since diagnosis, receipt of radiation. These covariates were not statistically significant and are therefore not shown in the table. Multiple imputation was used to assign household income values for the 13% of respondents who did not report it.

Involved. In bivariate analyses, a greater extent of partner involvement was significantly associated with a lower likelihood of patient receipt of surveillance CEA and all surveillance elements combined. The association between partner satisfaction with involvement and patient receipt of all elements of surveillance trended towards significance (p = 0.08). In multivariable analyses (Table 4), a greater extent of partner involvement was significantly associated with a reduced odds of patient receipt of all surveillance elements combined (OR 0.67, 95% CI 0.48–0.93). The association between a greater extent of partner involvement and reduced odds of patient receipt of surveillance endoscopy (OR 0.60, 95% CI 0.34–1.03) and CEA (OR 0.75, 95% CI 0.55–1.04) trended towards significance. Partner satisfaction with involvement trended towards significance for association with increased odds of patient receipt of all surveillance elements combined (OR 1.28, 95% CI 0.95–1.73).

Aware. In bivariate analyses, partner awareness of patients’ preferences for surveillance was significantly associated with an increased likelihood of patient receipt of surveillance endoscopy (p = 0.02). In multivariable analyses (Table 4), partner awareness of patients’ preferences for surveillance was significantly associated with an increased odds of patient receipt of surveillance endoscopy (OR 2.18, 95% CI 1.15–4.12) and trended towards significance for association with increased odds of receipt of all surveillance elements combined (OR 1.30, 95% CI 0.91–2.04).

## Discussion

Receipt of guideline-recommended CRC surveillance is important for the early detection of limited cancer recurrences, while there is still a chance for curative treatment. Guideline-recommended surveillance is a “package” of care; Patients should receive all elements of surveillance (cross-sectional imaging, endoscopy, CEA). In this dyadic survey study of survivors of Stage III CRC and their partners, we found varying levels of receipt of the individual elements of surveillance and sub-optimal receipt of guideline-recommended surveillance, with only 42% of patients reporting receipt of all elements. Partners in our study reported engagement across each domain, yet engagement varied by sociodemographic factors. Despite this engagement and contrary to our hypothesis we did not find an association between all domains of engagement and receipt of surveillance. Instead, the domains of partner engagement (informed, involved, aware) had differing effects on receipt of individual surveillance components and composite receipt of surveillance.

We did not find significant associations between partners’ perception of being informed about surveillance and patient receipt of surveillance. However, it is worth noting that we did not include an objective measure of partners’ factual knowledge about CRC surveillance, and that a perception of being informed is not equivalent to being informed. Further research is needed to determine whether providing partners with education about surveillance could be associated with improved receipt of surveillance among patients.

Our finding that a greater extent of partner involvement in surveillance was associated with a reduced odds of patient receipt of surveillance was surprising. Our data do not allow us to infer the direction of association. As has been shown in dyadic studies in prostate cancer [31], it is possible that some partners in our study value intensive surveillance more than patients. That is, partners only become greatly involved when patients are not receiving the most intensive surveillance, in an attempt to ensure that patients adhere to recommended care. Alternatively, it is possible that when partners are more involved than patients want them to be, patients react by avoiding recommended care. Previous studies have shown that family support does not always align with patients’ preferences [9], and that support that is perceived as nagging or critical can be a barrier to patients’ self-efficacy [32, 33]. Moreover, there may be mismatch between patients’ and partners’ perceptions of partners’ behaviors. A partner may perceive that they are very involved while the patient may perceive that the partner is not very involved or may prefer for the partner to be involved differently. Although we asked partners to report on various ways in which they are involved in surveillance, it is not possible to determine with certainty which activities the partners in our study are actually doing.

Our finding that partners’ satisfaction with their involvement was not associated with patients’ receipt of surveillance may also reflect that concordance around the concept of involvement may ultimately be more important in affecting patients’ behavior and clinical outcomes than partners’ satisfaction alone. This is reflected in our finding that increased partner awareness of patient preferences was associated with increased odds of patient receipt of surveillance. In our prior qualitative work with survivors of CRC and their partners [34], we found that patient and partner preferences for how the partner should engage in surveillance are not always concordant. Moreover, we found that partners often navigate this discordance and find ways to engage in surveillance that are acceptable to patients while also meeting their own needs as partners. The partners in our current study who reported greater awareness of patient preferences may represent dyads who have achieved such concordance of patient and partner preferences.

Taken together, our findings suggest that simply involving partners in the logistical aspects of surveillance care (e.g. instructing partners to attend surveillance appointments or take notes) may not be sufficient. Rather, ensuring that partners understand factual details about surveillance, helping partners improve their awareness of patients’ values and preferences for surveillance, and guiding partners in how to best communicate to achieve patients’ desired level of involvement could help improve patient receipt of surveillance. Existing interventions to improve communication between patients living with cancer and their family caregivers provide a model upon which to build future dyadic interventions in cancer care delivery [20]. Future interventions could assesses both patients’ and partners’ preferences for partner engagement in surveillance across all domains (informed, involved, aware) and then help dyads compare preferences and work through areas of difference to reach a mutual understanding of how the partner can best support the patient during surveillance. The provision of tailored feedback could help patients understand partners’ need to feel included, and help partners learn to effectively engage in surveillance in ways that are perceived as helpful by patients. As done previously, a web-based intervention would allow patients and their partners to use it together at a mutually convenient time [35].

There are several limitations of our study that warrant mention. Our findings were limited to patients with Stage III CRC recruited from an academic cancer center in Michigan, a rural oncology practice in Montana, and the state of Georgia and may not be generalizable to all patients. Nonetheless, we aimed for and achieved a broad distribution of patients. As with all survey studies, nonresponse bias was possible. When compared with patients whose partners responded to the survey, patients whose partners did not respond were more likely to be female and Black. We note that our patient response rate of 51% is comparable to or exceeds response rates of other large survey studies of survivors of CRC [36–38] and we also note our high response rate (73%) in partners. Additionally, as noted above, we could not assess directionality of the associations in our analyses. Our analyses did not include a full psychometric evaluation of the measures of partner engagement, which we adapted from a prior study of patients with breast cancer and their partners and family supporters [13]. However, we confirmed that the distribution of responses to the engagement measures and bivariate associations of engagement with partner age and education seen in our current study were similar to those seen in the prior breast cancer study. We also confirmed the internal consistency of the engagement domains using factor analysis and Cronbach’s α.

## Conclusion

We have shown that partners are engaged (informed, involved, and aware) in CRC surveillance, despite variations in extent of engagement by sociodemographic characteristics. While we found mixed results with regard to associations between domains of engagement and receipt of guideline-recommended surveillance, there remains room to leverage these domains and improve patient care. Dyadic interventions that capitalize on the positive aspects of partner engagement may help partners effectively engage in surveillance to improve patient care.

## Electronic supplementary material

Below is the link to the electronic supplementary material.

Supplementary Material 1

## Data Availability

The datasets generated during and/or analyzed during the current study are available from the corresponding author on reasonable request.

## List of abbreviations

CRC: colorectal cancer
CEA: carcinoembryonic antigen
SEER: Surveillance, Epidemiology, and End Results
CT: computed tomography
MRI: magnetic resonance imaging
PET: positron emission tomography
OR: odds ratio
CI: confidence interval
