# Supplementary material for: A dyadic survey study of partner engagement in and patient receipt of guideline-recommended colorectal cancer surveillance
Source: BMC Cancer. 2022 Oct 13;22:1060. doi: 10.1186/s12885-022-10131-3 (PMC9559022; doi:10.1186/s12885-022-10131-3)
Supplement: Supplementary file 1 — Supplementary Material 1 [file 12885_2022_10131_MOESM1_ESM.docx]

A Dyadic Survey Study of Partner Engagement in and Patient Receipt of Guideline-Recommended Colorectal Cancer Surveillance

*BMC Cancer*

Christine M. Veenstra, MD, MSHP^1^; Katrina R. Ellis, PhD^1^; Paul Abrahamse, MA^1^; Kevin C. Ward, PhD^2^; Arden M. Morris, MD, MPH^3^; Sarah T. Hawley, PhD^1^

^1^University of Michigan, Ann Arbor, MI; ^2^Emory University, Atlanta, GA; ^3^Stanford University, Stanford, CA

**Corresponding author:** Christine M. Veenstra, MD, MSHP, University of Michigan, 300 North Ingalls, NIB, Room 3A22, Ann Arbor, MI 48109; 734-272-6654; cveenstr@med.umich.edu

**Supplemental Figure 1:** Partner responses to each item within the engagement domains.

**Supplemental Figure 1a**. The proportion of partners who reported that they received enough **information** about each element of surveillance.

**Supplemental Figure 1b**. The distribution of partner responses to each item measuring **extent of involvement** in surveillance.

**Supplemental Figure 1c**. The distribution of partner responses to each item measuring **satisfaction with involvement** in surveillance. In creating the overall satisfaction with involvement score, the scoring for responses to the item “I would like to participate more in my partner’s follow-up care” was reversed such that an overall higher score indicates greater satisfaction with involvement.

**Supplemental Figure 1d**. The distribution of partner responses to the item, “How **aware** are you about your partner’s preferences for follow-up care?”
